# Measuring auditory cortical responses in *Tursiops truncatus*

**DOI:** 10.1007/s00359-021-01502-5

**Published:** 2021-07-30

**Authors:** Matt D. Schalles, Dorian S. Houser, James J. Finneran, Peter Tyack, Barbara Shinn-Cunningham, Jason Mulsow

**Affiliations:** 1 Present Address: Neuroscience Institute, Carnegie Mellon University, Pittsburgh, PA 15213 USA; 2 Biomedical Engineering, Boston University, Boston, MA 02215 USA; 3 National Marine Mammal Foundation, San Diego, CA 92106 USA; 4 US Navy Marine Mammal Program, Naval Information Warfare Center Pacific, San Diego, CA 92152 USA; 5 School of Biology, University of St Andrews, St Andrews, UK

**Keywords:** Auditory-evoked potential, Dolphin, EEG, Auditory cortical response

## Abstract

Auditory neuroscience in dolphins has largely focused on auditory brainstem responses; however, such measures reveal little about the cognitive processes dolphins employ during echolocation and acoustic communication. The few previous studies of mid- and long-latency auditory-evoked potentials (AEPs) in dolphins report different latencies, polarities, and magnitudes. These inconsistencies may be due to any number of differences in methodology, but these studies do not make it clear which methodological differences may account for the disparities. The present study evaluates how electrode placement and pre-processing methods affect mid- and long-latency AEPs in (*Tursiops truncatus*). AEPs were measured when reference electrodes were placed on the skin surface over the forehead, the external auditory meatus, or the dorsal surface anterior to the dorsal fin. Data were pre-processed with or without a digital 50-Hz low-pass filter, and the use of independent component analysis to isolate signal components related to neural processes from other signals. Results suggest that a meatus reference electrode provides the highest quality AEP signals for analyses in sensor space, whereas a dorsal reference yielded nominal improvements in component space. These results provide guidance for measuring cortical AEPs in dolphins, supporting future studies of their cognitive auditory processing.

**Supplementary Information:**

The online version supplementary material available at 10.1007/s00359-021-01502-5.

## Introduction

Bats and dolphins have sophisticated auditory systems for echolocation, making them of particular interest to comparative studies of auditory processing. Bats are an important model system in which invasive neurophysiological techniques have been utilized to study brain function during echolocation; for example, mapping cortical representation based on the time delay between outgoing echolocation pulses and returning target echoes in the auditory cortex (Bartenstein et al. 2014), which is an important calculation for prey capture and collision avoidance. In contrast, non-invasive auditory brainstem response (ABR) methods have been thoroughly developed to study dolphin hearing at early response latencies, on the order of 1–10-ms post-stimulation (for a review, see Supin et al. 2001). These methods have been increasingly used to study the processing of the animal’s own outgoing biosonar signal and incoming echoes, although the responses are limited to subcortical auditory processing. Studies of longer latency potentials, such as auditory cortical responses (ACR), are necessary to provide insights into the cognitive processes that these animals employ to make sense of the complex acoustic mixtures they hear during echolocation, and facilitate comparative work with human cognitive study.

There are a number of technical challenges to collect ACRs from free-swimming dolphins (in addition to the relative paucity of data on the dolphin ACR as compared to the ABR). Among these challenges are the limitations to sensor placement in free-swimming animals (i.e., not restrictive to swimming or feeding, or the ability to deploy in autonomous tags), and the elimination of movement or extraneous physiological potentials that are likely to interfere with the lower frequency spectral content of the ACR. Parallel work in human electroencephalography (EEG) has yielded significant advances in recording and processing techniques to address parallel challenges in less than ideal experimental conditions, and could be applied to dolphin AEP experiments.

In the present study, we manipulated three parameters central to developing ACR recording methods with free-swimming dolphins: the placement of reference electrodes to identify positions that produce robust results while minimizing interference with dolphin swimming; the application of an offline, low-pass filter akin to those used in human event-related potential (ERP) studies which also serves to pre-process data for spatial filtering; and the use of a spatial filter to separate brain signals from other noise. A summary of relevant literature on these topics is provided below. While prescribing a standard protocol for recording is beyond the scope of this work, our goal was to provide recommendations for acquisition of evoked potentials that build on the existing body of dolphin auditory processing studies, including the more extensive ABR research.

### Response latency and electrode placement

One major question in interpreting non-invasive recordings is how the placement of an inverting, or reference electrode, and the non-inverting electrodes interact to affect the observed response. Electric signals recorded at different locations reflect a different summation of underlying neural generators. Some insight is gained from early reports of auditory-evoked cortical recordings from dolphins that used invasive electrodes, and the later attempts to link them to skin surface recordings. However, resulting comparisons between studies are complicated because of important differences in methodology, including where electrodes were placed and what reference location was chosen.

Past studies of ACRs can be broadly categorized as relying on data from electrodes implanted in brain tissue (Bullock and Ridgway 1972; Supin et al. 1978), implanted at the skull surface (Seeley et al. 1976; Ridgway 1980; Woods et al. 1986), or placed at the skin surface (Popov and Supin 1986; Hernandez et al. 2007). These different recording montages are summarized in Table 2. Without directly accounting for polarity differences across studies, the most commonly reported ACR has two prominent component peaks of alternate polarities (Bullock and Ridgway 1972; Ridgway 1980; Popov and Supin 1986; Supin et al. 2001). The first component peak latency reported between 18 and 30 ms post-stimulus-onset, and the second component peak latency between 24 and 100 ms (see Table 1). These resemble the component structure of a human N1–P2 complex (Picton 2011), albeit with significantly shorter latencies than humans. Intracranial recordings identified at least three different types of ACRs, categorized by latency (Supin et al. 1978). These different ACRs exhibited peak cortical responses as early as 9 ms and as late as 60 ms, and showed topographic differences in both latency and peak component structure. Two subsequent studies of ACRs in dolphins manipulated stimulus probability to elicit an auditory oddball effect in response to infrequently occurring acoustic stimuli (Woods et al. 1986; Hernandez et al. 2007). Both reported prominent ACR peaks in the latency range that generally describes a dolphin cortical N1–P2. However, only Hernandez et al. (2007) reported oddball effects within this range; Woods et al. (1986) reported them at longer intervals more homologous to human experiments (Squires, Nancy et al. 1975). While the latency range of intracranial recordings confirms the prospective cortical origin of the earliest ACR response latencies, the differences in reported latency and peak structure between studies may be due to differences in the recording depth and anterior–posterior and medial–lateral electrode positions.

**Table 1:** Peak latency from stimulus onset (in milliseconds) for ACR peaks across past studies, irrespective of peak polarity

Study	Peak 1	Peak 2	Peak 3
Bullock and Ridgway (1972)	22	40	–
Seeley et al. (1976)	100	200	–
Supin et al. (1978)	9–15	20–30	50–60
Ridgway (1980)	30	100	–
Woods et al. (1986)	25	200	550
Popov and Supin (1986)	18–19	24–30	–
Hernandez et al. (2007)	25–30	40–60	64–86

Some of the earliest reports used intracranial recordings from multiple electrode sites. Bullock and Ridgway (1972) implanted depth electrode arrays to identify responses from brainstem and cortex. Popov and Supin (1976) used a movable monopolar electrode to locate cortical tissue that produced an evoked response to click stimuli. This approach was extended to generate an initial mapping of auditory cortices (Ladygina and Supin 1977; see also summaries by Supin et al. 1978; Bullock and Gurevich 1979). Moveable needle electrodes placed in the skin at a depth of 2–3 mm found a maximal magnitude ACR along the midline, at a position 20 cm posterior to the blowhole (Popov and Supin 1986); the polarity of the signal flipped as the electrode moved anteriorly, toward the blowhole, from the midline position of maximal magnitude. Compared to intracranial data localized to the suprasylvian gyrus, this suggests that the dipole of the ACR source is oriented in a rostroventral–dorsocaudal direction (Supin et al. 2001). Most recently, ACRs were obtained from skin surface sites using gold cup electrodes embedded in suction cups, with the non-inverting electrode placed 4-cm lateral and posterior to the blowhole (Hernandez et al. 2007). This electrode position is similar to those commonly used for recording ABRs (Supin et al. 2001).

**Table 2:** EEG recording parameters from previous ACR studies

Study	Electrode depth	Electrode location	Reference location	Hi-pass filter (Hz)	Low-pass filter (Hz)
Bullock and Ridgway (1972)	Cortex	posterolateral temporal	Unspecified	2	$10,000^{\mathrm{a}}$
Seeley et al. (1976)	Skull	vertex	Anterior dorsolateral head	1	30
Supin et al. (1978)	Cortex	multiple*	Unspecified	–	–
Ridgway (1980)	Skull	vertex	Temporal	–	–
Woods et al. (1986)	Skull	vertex	Snout/mastoid	1	$3000^{\mathrm{b}}$
Popov and Supin (1986)	Subdermal	multiple*	Unspecified$^{\mathrm{c}}$	0.3	3000
Hernandez et al. (2007)	Skin	4 cm posterior and	Meatus	1	100
		Lateral to blowhole			

*Details elaborated in “Response latency and electrode placement”.

$\mathrm{a}$Author stated band-pass was either 10–5000 or 2–10,000 Hz depending on session.

$\mathrm{b}$ Author described a digital sample rate of 213 Hz, with an anti-aliasing filter of − 3 dB at 100 Hz.

$\mathrm{c}$ The reference was likely placed on either the the pectoral or dorsal fin (Supin, personal communication, 178th meeting, Acoust. Soc. Am., San Diego 2019)

The reference electrode locations summarized in Table 2 detail the wide variation of reported recording montages. The relative positions of the reference and non-inverting electrodes can influence the magnitude of an evoked potential, particularly if they are situated on opposite sides of the source dipole. For a dolphin ABR, a reference placed over the temporal, mastoid, or meatus markers could amplify the response for a non-inverting electrode placed on the dolphin’s dorsal midline, such as near the blowhole. However, given proximity to brain tissue, these references could also introduce potentials corresponding to other neural processes or systems. Because the observed response is the sum of multiple underlying sources, observed AEP peak amplitudes and latencies could shift when other potentials are added; more neutral references, such as placed over a fin or snout, may reduce such effects. Due to the inconsistent reporting and varying processing techniques in published dolphin ACR work, it is not entirely clear how the interaction of reference and non-inverting electrode placement affected previously reported ACRs. However, the interaction of reference and non-inverting electrode recording locations can be addressed empirically through controlled manipulation of reference electrode placement. A better understanding of this interaction would aid the interpretation and synthesis of the previous ACR literature.

### Filtering

There is no standardized processing pipeline for measuring cortical AEPs from dolphins, and it is possible that the differences in reported ACR latencies are at least partially due to differences in filtering. Past studies have not consistently reported the filter parameters used, nor necessarily maintained consistent parameters throughout the study (see Table 2). Recording EEG from awake, unrestrained dolphins possess practical challenges, and researchers often face a dilemma to balance practical and experimental constraints. It might be desirable in some experiments to use more restrictive filter settings than a priori theoretical assumptions based on response latency would suggest.

In humans, the band-pass filter settings are well characterized to isolate the ABR (30–3000 Hz), mid-latency (5–500 Hz), and long-latency (1–30; 0.1–15 Hz) AEPs, with the latter generally understood to be cortical in origin (Picton 2011). In dolphins, the ABR mostly exhibits energy between 600 and 2000 Hz (Supin et al. 2001), and can be reliably recorded with band-pass filter settings of 300–3000 Hz that filter out both physiologically derived noise, such as myocardial and motor potentials, and 50–60-Hz line noise (Finneran et al. 2013). Widely varying reported cortical response latencies for dolphins (Table 1), which straddle the human mid- and long-latency ranges, make the ideal filter parameters are for a given experiment less clear. One solution is to record with the widest possible filter passband setting, such as the 0.3–3000-Hz band-pass reported by Popov and Supin (1986), and then digitally filter offline. Depending on the experiment, periodic movement or motor potentials could cause the recording to exceed the maximum allowable voltage of the analog-to-digital converter, and in practice may require a more restrictive high-pass filter, such as 1–3 Hz. However, long-latency cortical components, like the human auditory oddball P3 that is evoked by unexpected stimuli, contain energy below 1 Hz. Even a high-pass filter that cuts off at 1 Hz (e.g., attenuates 3–6 dB relative to the passband response) would attenuate such responses in humans (Duncan-Johnson and Donchin 1979; Tanner et al. 2015), and later cortical components in dolphins, like the P550 (Woods et al. 1986). To help isolate cortical responses from other sources of brain activity, it may be prudent to low-pass filter the data, as well. Many studies summarized in Table 1 suggest the earliest response latency recorded outside of neural tissue to be approximately 20 ms or more. In this instance, a filter such as a 50-Hz low-pass should preserve cortical AEP components of interest.

When electrodes are spaced close enough that an evoked signal (and any artifacts) produces correlated responses across electrodes, spatial filters such as Independent Component Analysis (ICA) have proven useful for source decomposition. Specifically, ICA offers a method for artifact removal as well as a way to isolate neural signals from background noise (e.g., Jung et al. (1998)). This approach works best when the artifacts arise from some physiological source with stable spatial patterns on the skin surface (Onton et al. 2006). ICA assumes that the sources of electrical signals are independent, e.g., a muscle controlling blowhole action is a discrete source that causes a distinct pattern different from those caused by post-synaptic potentials in the auditory cortices. By applying weights to the EEG signals, they can be summed into components, and ICA tunes the weights to maximize independence between the summed component activity (Makeig and Onton 2012). Once constituent components are computed, components that code artifacts can be identified and rejected; the remaining (signal) components can then be back-projected to sensor space to reconstruct the signal without the influence of the artifacts. Alternately, if an independent component (IC) contains sufficient information from a neurophysiological process of interest, such as an AEP, it can be treated as a representative source of that underlying neural generator (Nunez et al. 2019). This approach introduces the concept of performing analysis on components of interest in source space, which is functionally similar to performing conventional analysis on recorded EEG channels in sensor space. As a component can be described as a spatial filter, when applied to EEG channels, its resulting time series is often referred to as component activations. Of interest to the present study is whether a small array of non-inverting electrodes may enable the use of ICA to address the challenges of conventional filtering in the study of the dolphin ACR by helping to separate the auditory response from artifacts.

## Methods

### Subject

All data were collected on a male dolphin, OLY, aged 35 years and with a mass of 185 kg. He exhibited high-frequency hearing loss (upper frequency hearing limit of 70 kHz defined by a hearing threshold exceeding 120 dB re ${1}\,{\upmu \hbox {Pa}}$), but all tones used in the present study were detectable based on auditory steady state response hearing thresholds (Strahan et al. 2020). He was housed in netted enclosures (9$\,\times \,$9 to 9$\,\times \,$18 m) within San Diego Bay, California. The study followed a protocol approved by the Institutional Animal Care and Use Committee at the Naval Information Warfare Center Pacific and the Navy Bureau of Medicine and Surgery, and followed all applicable U.S. Department of Defense guidelines for the care of laboratory animals.

### Stimulus and delivery

The dolphin voluntarily beached onto a padded mat and rested under shade for the duration of the recording sessions. A jawphone, consisting of a hydrophone (ITC-1042) embedded in a degassed silicone rubber suction cup (Rhodia V-1065), was positioned over the pan region of the dolphin’s left mandible in accordance with ANSI/ASA S3/SC1.6 (Accredited Standards Committee 2018). As the standard cites an estimated distance of 15 cm between the pan region and the ipsilateral auditory bulla for this species, the jawphone transmitter was calibrated underwater, 15 cm from a receiving hydrophone (Reson TC4013). Stimuli were presented at a sound pressure level (SPL) of 120 dB re ${1}\,\upmu \hbox {Pa}$ 300-ms pure tones with a 20-kHz center frequency and linear 5-ms rise/fall times, and were delivered with a pseudo-random temporal jitter, linearly distributed between 1000 and 1500 ms. The stimuli were generated in LabVIEW software (National Instruments, v.2018) at 16-bit resolution with a 512-kHz update rate. An NI PXI-6251 data acquisition card (National Instruments, Austin, TX) converted the digital signal to an analog voltage (16-bit resolution, 512-kHz update rate) that was band-pass filtered from 5 to 200 kHz (Krohn-Hite Corporation, Brockton, MA).

### Electroencephalographic recording

**Fig. 1:**
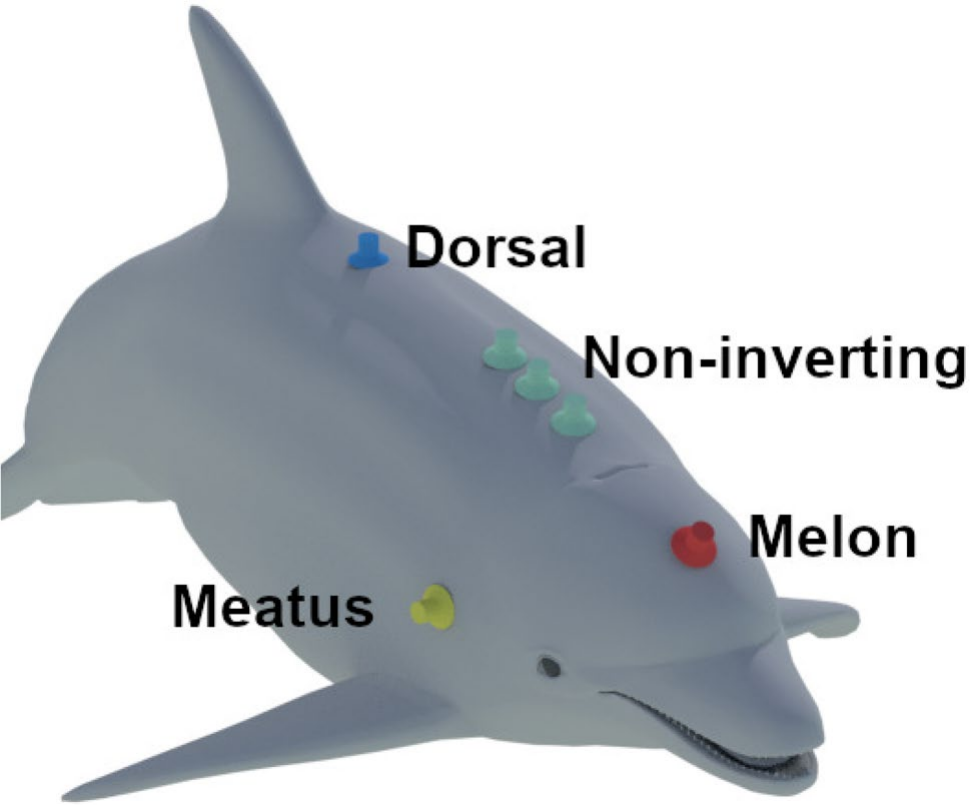
Placement of midline non-inverting electrodes (green) relative to the placement of the reference: over the meatus (yellow), on the melon (red), and anterior of the dorsal fin (blue)

Both non-inverting and reference electrodes (Natus 10-cm gold cup) were embedded in silicone suction rubber cups (Rhodia V-1065) and placed on the dolphin with a small amount of conductive paste spread within the cup electrode. Three non-inverting electrodes were placed along the dorsal midline 10, 20, and 30 cm posterior to the caudal lip of the blowhole. Previous experiments suggested that placement 20 cm posterior to the blowhole on the midline should yield the maximal amplitude for an ACR (Supin et al. 2001; Popov and Supin 1986), and further suggested that neighboring electrodes on the dorsal midline should contain mutual information, which is relevant to ICA decomposition. The single inverting, or reference, electrode was either positioned over the external auditory meatus (right side), the midline just anterior to the dorsal fin (as described in ANSI/ASA S3/SC.6 (Accredited Standards Committee 2018)), or centered on the melon/forehead (see Fig. 1).

We used a three-electrode montage centered at the vertex, which we refer to as “vertex placement.” We hypothesized that the site-specific information provided by this montage along the longitudinal axis might inform the application of a spatial filter to further isolate the ACR from physiological noise, such as heart or skeletal muscle contractions. The midline positioning should provide coverage of local voltages produced by the superior auditory processing areas (Supin et al. 1978). However, as there are few, if any, neuroanatomical markers available from the exterior of the animal (Bullock and Ridgway 1972), the comparison of results from recordings made inside the skull with those closer to the surface of the head need to rely on functional properties of the responses rather than superficial similarities in the recording site.

EEG signals were amplified using three Grass IP511 biopotential amplifiers (10,000$\times$ gain, band-pass filter of 1–3000 Hz). The recording was grounded with an electrode placed in the seawater near the dolphin. The signal was digitized at a 10-kHz sample rate using the same PXI-6251 card that controlled the stimulus output. Additionally, the outgoing stimulus voltage was recorded as a fourth analog input channel, allowing us to synchronize analysis to event onsets.

### Analysis

EEG signals were analyzed using EEGLAB toolbox (v14.1.2) for Matlab (Delorme and Makeig 2004). Data for the unfiltered condition were segmented into epochs from −100 to 1000 ms relative to stimulus onset. The mean voltage value from the pre-stimulus period (−100 to 0 ms) was subtracted from the post-stimulus epoch voltage to baseline correct each epoch. For the filtered condition, prior to epoch segmentation, EEG data were downsampled to 1000 Hz with EEGLAB’s ’resample’ function, which includes an anti-aliasing filter, and then band-pass filtered from 1 to 50 Hz (3300 point FIR with zero phase).

An adaptive voltage threshold was used to remove high-amplitude artifacts. The threshold was initially set at ${100}\upmu \hbox {V}$, and if more than 20% of the epochs were removed, the algorithm raised the threshold in ${25}\upmu \hbox {V}$ steps until fewer than 20 but not fewer than 1% of the epochs were rejected. Visual inspection was used to ensure that no artifacts were overlooked by the automated procedure, and any remaining non-stationary artifacts (Jung et al. 2000) were removed to aid the ICA decomposition. This left approximately 1100–1150 epochs per electrode montage. To normalize the epoch numbers in each dataset, 1100 epochs were pseudorandomly selected from the remaining data in each set, without replacement. Independent components were extracted using the EEGLAB ’runica’ implementation of the ’Infomax’ algorithm (Delorme and Makeig 2004).

The signal-to-noise ratio (SNR) were estimated for each condition [non-inverting electrode (3) $\,\times \,$ reference electrode placement (3) $\,\times \,$ filter (2)] and reported for both sensor and source spaces. Based on the process described by Elberling and Don (1984), and modified for the latency range of a cortical response, a signal term was estimated as the root-mean-square of the averaged AEP waveform from 1- to 100-ms post-stimulus onset. The noise term was estimated from the square root of the variance of a single point randomly selected within the AEP signal time-window across all epochs, divided by the number of epochs. The SNR was estimated as the square root of the ratio of the signal squared divided by the noise squared minus one (see Finneran et al. 2019 for equations) reported in dB (i.e., the SNR level, which will subsequently be referred to as SNRL). Only electrodes or components with SNRL > 6 dB were included in subsequent AEP analysis (Mulsow et al. 2020).

AEP amplitudes were reported for an early cortical sensory complex using the same temporal windows as Hernandez et al. (2007), where P50 was the vertex-positive peak occurring between 40- and 60-ms post-stimulus onset, and N75 was the vertex-negative peak occurring between 65 and 85 ms. As preliminary analysis suggested the polarity of our measures was inverted relative to previous reports (see discussion), we refer to these time windows as N1 and P2, respectively. N1 and P2 amplitudes were reported both as the mean voltage and the peak magnitude voltage within each window. Peak-to-peak values were calculated as the difference between the N1 and P2 peak amplitudes. As ICA sources may invert polarity relative to sensor-space recordings, the negative and positive peaks were constrained to their respective time windows, and calculated as the maximum absolute voltage value.

ICA decomposition has a stochastic element, and as there are few published data to validate ICA-derived AEPs, component reliability for the dorsal-referenced component was tested using a split-half comparison method (Groppe et al. 2009). Specifically, the full data set was split into odd and even numbered epochs, and ICA performed separately on each of the halves. To determine the similarity of the resulting analyses, pairwise comparisons were made between each half and the full data set by calculating the cosine distance between component weights, and the residual variance between component activations.

## Results

### Sensor space

Highest SNRLs were found from dorsal head surface non-inverting electrodes 20–30 cm posterior to the blowhole and referenced to the meatus, and the 20-cm electrode posterior to the blowhole and referenced to the melon (see Table 3). These were the only electrode-reference pairs that exceeded the 6-dB threshold supporting further analysis. For these pairs, a low-pass filter at 50 Hz increased the SNRL by approximately 1 dB. The lower SNRL values for the dorsal reference were most likely a result of large electrocardiogram (ECG) contamination (see supplemental Fig. 1a). These ECG artifacts, which presented as high positive amplitude transients spread heterogeneously through the stacked time series, were greatest for the dorsal reference, but were also present in melon and meatus-referenced recordings. When referenced to the meatus or melon, which is anterior to the non-inverting electrodes, the ECG contamination increased across the non-inverting electrodes in the posterior direction (i.e., as non-inverting electrode placement moved closer to the heart).

**Table 3:** Sensor-space signal-to-noise level estimates (dB): reference electrodes by non-inverting electrodes (labeled by distance posterior to blowhole)

Reference	50-Hz Low-pass filtered			Unfiltered		
	$\ 10\ cm$	$\ 20\ cm$	$\ 30\ cm$	$\ 10\ cm$	$\ 20\ cm$	$\ 30\ cm$
$Dorsal$	4.5 (−6.7)	3.9 (−7.3)	5.6 (−5.6)	4.6 (−6.6)	4.0 (−7.2)	5.7 (−5.5)
$Melon$	5.9 (−4.2)	8.3 (−1.8)	5.3 (−4.8)	5.1 (−5.0)	7.9 (−2.2)	5.5 (−4.6)
$Meatus$	4.7 (−6.7)	10.0 (−0.4)	10.4 (0)	4.7 (−5.7)	8.8 (−1.6)	8.5 (−1.9)

Difference from max reported SNRL value across all conditions in parenthesis

**Fig. 2:**
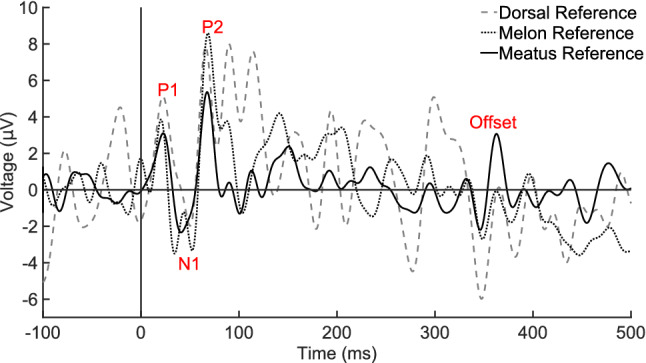
Low-pass filtered (50-Hz) ACR from the non-inverting electrode 20 cm posterior of the blowhole and referenced to three different locations. AEP components are labeled P1, N1, P2, and offset response, which was most prominent in the meatus-referenced average

**Fig. 3:**
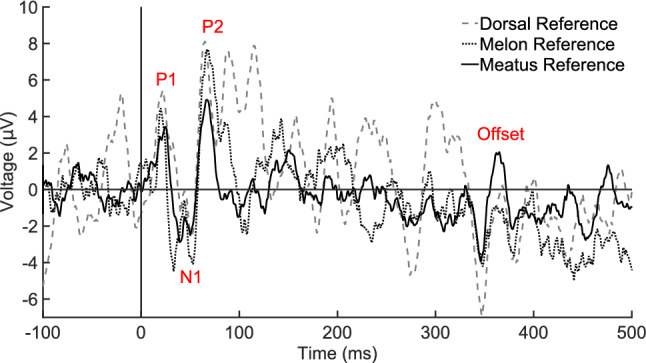
Unfiltered ACR from the non-inverting electrode 20 cm posterior of the blowhole and referenced to three different locations. AEP components are labeled P1, N1, P2, and offset response, which was most prominent in the meatus-referenced average. Positive voltage is plotted as up on the Y-axis

For meatus- and melon-referenced recordings, the time course of the ACR in the first 100-ms post-stimulus onset was similar to previous reports, albeit with an inverted polarity (Hernandez et al. 2007). This is likely explained by the location of the non-inverting electrode, a point that is elaborated in the discussion section. The averaged waveform (Fig. 2) shows a P1 around 25 ms latency, an N1 at approximately 50 ms, followed by a P2 at 75 ms. The P1–N1–P2 component and latency structure was similar for both dorsal and meatus montages, while the melon-referenced N1 contained a bifurcated peak. The unfiltered ACR also indicated a bifurcated N1, but with a different period between the local minima that was shorter for the meatus-referenced average than for the melon (Fig. 3). Given the periodicity of background noise in the melon-referenced recording, this bifurcation may be an artifact. In addition, the meatus-referenced recording, but not the dorsal or melon, contained a prominent offset response of similar latency and peak structure as the N1–P2, but occurring 350–375-ms post-stimulus onset (Fig. 2).

The peak-to-peak amplitude for N1–P2 (Table 4) was largest for the melon reference; as the meatus reference showed larger SNRL than the melon, this suggests that the melon reference is susceptible to higher amounts of noise. Specifically, given the observed peak values, the expected mean N1 and P2 values were smaller than expected. We thus undertook a post hoc analysis using a temporal window shifted 8 ms earlier than that originally used by Hernandez et al. (2007). This shifted analysis increased the mean values at the 20-cm electrode site for both melon and meatus montages, with minimal effect on the peak magnitudes for N1 and P2 (see supplementary Tab. 1).

**Table 4:** Sensor-space ERP N1-P2 component magnitude and peak-to-peak amplitude for reference montage and non-inverting electrode (distance from blowhole) pairs ($\upmu \hbox {V}$)

Electrodes	Filtered					Unfiltered				
	*N*1	*N*1	*P*2	*P*2	$Peak-$	*N*1	*N*1	*P*2	*P*2	$Peak-$
	Peak	Mean	Peak	Mean	Peak	Peak	Mean	Peak	Mean	Peak
$Melon{\text {-}}20\, cm$	3.34	1.71	8.60	5.74	11.94	4.07	1.75	7.69	5.30	11.76
$Meatus{{\text {-}}}20\, cm$	2.35	0.95	5.36	2.37	7.70	2.89	1.20	4.92	2.01	7.78
$Meatus{\text {-}}30\, cm$	1.16	0.792	8.48	5.57	9.63	2.05	0.28	7.24	4.35	9.29

### Source space

**Fig. 4:**
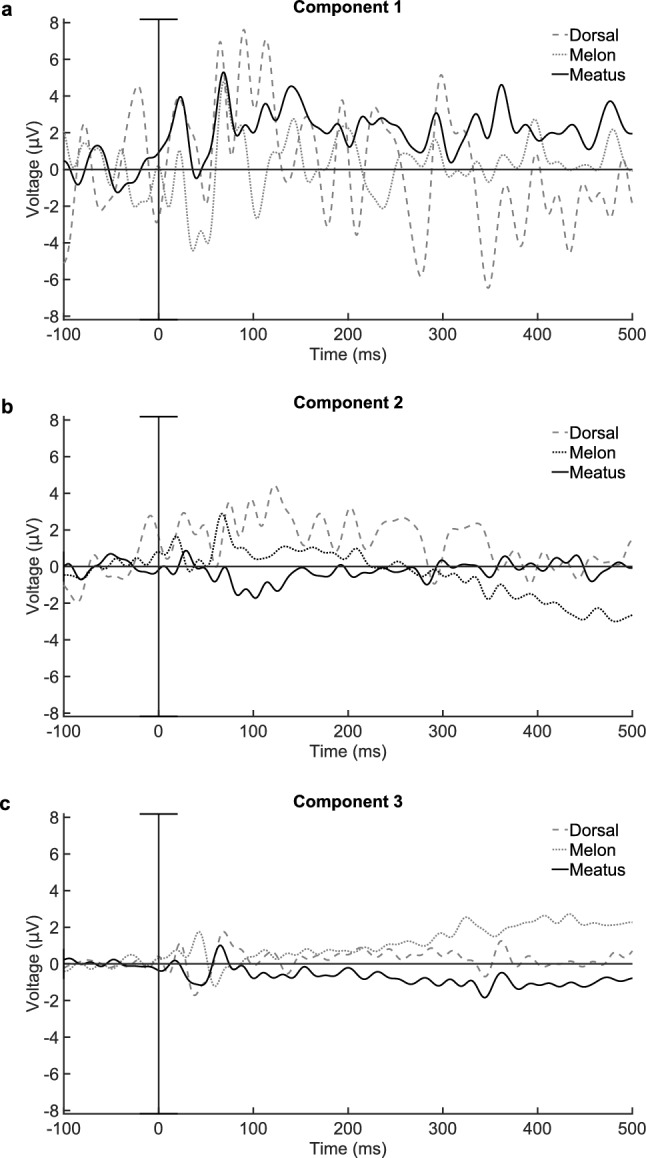
Event-related potentials from three independent components across all three reference montages. Component three most closely resembles the AEP structure previously reported by Hernandez et al. (2007)

**Fig. 5:**
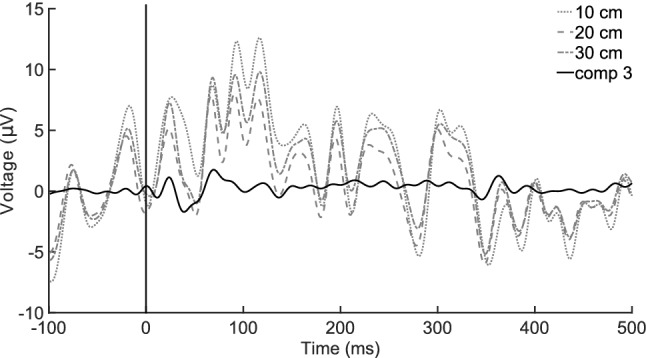
Event-related potentials comparing sensor space with the third independent component from the dorsal reference montage. The potential for the 20-cm non-inverting electrode is the same data represented in Fig. 2

Ideally, an ICA algorithm can separate a noise source, such as the heartbeat, from an independent signal source such as the AEP. If both evoked responses and consistent biological noises are significant in EEG recordings, ICA components associated with the evoking sound should have a relatively large SNRL value, and components associated with biological noise should have an apparently small SNRL value. Compared with sensor-space data, the components derived from the dorsal montage exhibited this pattern: the first ranked independent component appears to represent heartbeat contamination with periodic large amplitude deflections not time-locked to the stimulus^1^, while the third ranked IC predominantly contains the AEP with minimal noise. Table 5 gives the SNRLs for the ICs for the different reference montages. The largest SNRL among all conditions was in the third ranked component from the dorsal-referenced recording, in both filtered and unfiltered data sets. Melon and meatus-referenced recordings also showed relatively strong SNRL values for their third ranked components; however, the largest SNRLs in the filtered datasets were for the first ranked IC from the meatus-referenced data and for the second ranked IC in the melon-referenced data. Visual inspection of the evoked potentials represented by these components (Fig. 4) suggested that the top ranked component described a primarily noise artifact; however, in the case of the meatus, the first ranked IC also contained some of the AEP. Across the three montages, the third ranked IC contained a visually apparent onset and offset response with minimal background noise that followed the time course of the sensor-space ACR from the 20-cm meatus montage. This time course for onset response also followed that previously reported by Hernandez et al. (2007). Even though other ICs had relatively large SNRLs during the 0–100-ms time window, subsequent ERP measures were reported only for the third ranked IC across each reference montage (Table 6). Figure 5 compares the sensor-space recordings with high noise levels and the third ranked IC from the dorsal reference montage.

**Table 5:** Component-space signal-to-noise level estimates (dB): components by reference electrodes

Reference	Filtered			Unfiltered		
	$Comp\ 1$	$Comp\ 2$	$Comp\ 3$	$Comp\ 1$	$Comp\ 2$	$Comp\ 3$
$Dorsal$	2.2 (−9)	5.8 (−5.4)	11.2 (0)	2.0 (−9.2)	5.8 (−5.4)	10.1 (−1.1)
$Melon$	5.6 (−4.5)	10.1 (0)	8.5 (−1.6)	5.8 (−4.3)	6.9 (−3.2)	7.8 (−2.3)
$Meatus$	10.4 (0)	5.7 (−4.7)	10.0 (−0.4)	8.5 (−1.9)	4.1 (−6.3)	8.9 (−1.5)

Difference from max reported SNRL value across all conditions in parentheses

**Table 6:** ERP N1–P2 component magnitude and peak-to-peak amplitude for the third independent component ($\upmu \hbox {V}$)

Reference	Filtered					Unfiltered				
	*N*1	*N*1	*P*2	*P*2	$Peak-$	*N*1	*N*1	*P*2	*P*2	$Peak-$
	Peak	Mean	Peak	Mean	Peak	Peak	Mean	Peak	Mean	Peak
$Dorsal$	1.65	0.83	1.76	1.28	3.40	1.69	0.82	1.92	1.27	3.61
$Melon$	1.75	0.39	0.77	0.28	2.52	1.48	0.24	0.72	0.32	2.20
$Meatus$	1.16	0.69	1.02	0.18	2.18	1.07	0.58	0.92	0.18	1.98

The component reliability measures indicated that the components identified as exhibiting AEP properties shared small cosine distance between their weights, and likewise small residual variance among their activations (see supplementary Tab. 2 and 3). The visual similarity between halves and the full dataset for the third IC is depicted in the AEP, which suggests reliability of the ICA decomposition (supplemental Fig. 2). The smallest cosine distances and residual variances were observed for the first component, which represented the majority of the myocardial and other noise sources.

## Discussion

Through a series of EEG experiments that manipulated reference electrode placement and application of offline frequency domain and spatial filters, we observed AEPs with a peak-component structure containing a prominent N1 at approximately 50 ms, and P2 at approximately 75 ms. Accounting for a polarity inversion attributable to electrode montage, this component structure closely resembles that reported by Hernandez et al. (2007). Application of a 50-Hz low-pass filter during EEG pre-processing slightly increased the SNRL, but choice of reference electrode location had a larger effect on the noise in the responses. For analysis in sensor space, the best SNRL for the auditory cortical response was obtained at the non-inverting electrode 20 cm posterior to the blowhole on the midline referenced to the meatus. In component space (from ICA), the greatest SNRL was obtained using the dorsal reference montage.

### Recording and pre-processing considerations

We used SNRL measures to compare the fidelity of AEPs over the presumed cortical response latency window recorded from different electrode montages and pre-processing parameters. Many ERP studies estimate signal strength based on peak amplitudes, and rely on a baseline correction, or subtraction of a pre-stimulus voltage from the remaining epoch window, to effectively center the voltage to zero at the time of stimulus onset. Given that our recordings included significant artifact contamination during the pre-stimulus baseline for some conditions, it seemed prudent to use another method to assess AEP fidelity. We adopted a method commonly used in human ABR analysis (Elberling and Don 1984), which was previously applied to dolphin ABR measurements using a similar recording paradigm to that used here (Finneran et al. 2019). By the SNRL metric, the meatus-referenced montage produced the highest fidelity AEP. While not addressed here, follow-up work could make use of this SNRL metric to estimate the trade-off between number of epochs and SNRL for mid- or long-latency AEPs to help characterize what might be an optimal number of epochs for ACR experimental sessions. As previously described by Popov and Supin (1986), somewhere between 300 and 3000 epochs was appropriate for most applications. However, their experiment was performed with needle electrodes with an insertion depth of 2–3 mm, which yields recordings with potentially higher SNR than electrodes applied to the skin surface.

One of the parameters we varied was the location of the reference electrode. Animals in two prior studies were suspended in a stretcher in a shallow pool (Woods et al. 1986; Popov and Supin 1986). This allowed the use of electrode locations, such as on fins, which could present additional complications in the study of free-swimming dolphins in underwater environments with an EEG system. Since our long-term goal is to undertake such studies, we did not test these locations. Likewise, we did not consider placing electrodes on the rostrum. As we want to test awake, unrestrained animals who are periodically fed with fish throughout the recording session, attachments to the rostrum are likely to fail. These considerations guided our selection of the three reference electrode locations we tested. While the meatus position was lateralized similarly to a mastoid reference in humans, the dorsal and melon sites were at midline, which should avoid a lateral bias in the measures.

Application of a digital 50-Hz low-pass filter did not significantly affect amplitude measures of the ACR. While it nominally increased the estimated SNRL by about 1 dB, it did not seem to affect the peak to peak amplitude of the N1–P2. This supports the use of such restrictive filtering in future experimental protocols where there is the potential for contamination by higher frequency noise. It also supports the use of such a filter as part of the pre-processing for ICA.

ICA decomposition produced at least one component for each of the three reference montages that contained enough information to represent an AEP source relatively devoid of noise contamination. As ICA maximizes the independence of shared information between components (Makeig et al. 2004), it will inevitably affect the underlying variance across epochs in the component activations. This could bias the noise term in the SNRL estimates, thus inviting caution in interpretation of the relative component SNRL values. For example, the first and third components from the meatus-referenced montage have roughly equivalent SNRL values; however, the average waveform for component one contains much larger background noise than the average waveform for component three (see Fig. 4A, C). The larger noise term in component one is offset by a larger signal term, as well. Both components appear to represent a portion of the true AEP. This suggests that the decomposition could be even more successful at isolating the AEP with a larger array of electrodes. Given that we used only three non-inverting electrodes, ICA has limited utility and cannot adequately decompose all of the diverse underlying neural and noise sources in the recordings into a full-rank set of three components. However, even in this limited capacity with a small electrode array, the ability to separate an AEP from background noise shows significant potential for application to future dolphin studies where recording conditions are less than ideal. With ICA separation of the ECG component, the dorsal-referenced montage produces better results for a relatively clean AEP component. Thus, it may be that a reference location even farther away from the AEP generator tissue than the meatus or melon references could effectively serve as a more neutral reference in that it is less likely to contain AEP information.

It can be difficult to interpret ICA components, and they can yield ambiguous results, such as the polarity of activations and their resulting ERPs. As visible in the melon-referenced trace in Fig. 4C, the polarity of a component activation can flip relative to the information it represents from sensor space. As ICA may converge on a solution that has two possible polarity values for the weight matrix, the sign of the sensor-space data is represented by the product of both the component activation and its inverse weight matrix for back projection (Onton et al. 2006).

ICA solutions are not found from a closed form computation, but instead are estimated from a stochastic process. Therefore, the same (or very similar) data can produce different solutions. To deal with this uncertainty, we employed a reliability algorithm using split-half comparisons (Groppe et al. 2009). While there is no objective criterion for determining the reliability of an ICA solution, if disjoint data sets produce similar solutions, it bolsters faith that the decompositions are capturing key aspects of the data. This approach is strengthened if solution similarity, captured as the cosine distance and residual variance measures, is greater for data sets that should contain the same signal and noise components (e.g., both representing statistically identical AEP or ECG activity) than for data sets that should differ (e.g., comparing components derived from AEPs and ECGs). If the former are smaller than the latter, it supports the view that the decomposition is identifying functionally relevant and reliable components of the measurements.

### Interpretation of auditory-evoked potentials

Our ACR results resemble those reported by Hernandez et al. (2007), but with opposite polarity. This difference is likely explained by differences in the locations of the non-inverting electrodes on the head surface in the two studies. Supin et al. (2001) described a shift in polarity of the ACR moving along the anterior–posterior axis, with a polarity flip approximately 5–10 cm posterior to the caudal lip of the blowhole. Hernandez et al. (2007) reported their non-inverting electrode was 4 cm posterior to the blowhole; in contrast, we used montages with electrodes 10, 20, and 30 cm posterior to the blowhole, which supports this anterior–posterior axis change in polarity.

Our results found the greatest SNRL in sensor space for the 20-cm non-inverting electrode, consistent with prior reports that the largest magnitude ACRs are recorded 20 cm posterior to the blowhole (Popov and Supin 1986). However, differences in electrode depth and inverting electrode placement can also influence SNRL and ACR magnitude measures. To address this directly, one could perform a follow-up multi-electrode study using non-inverting recording sites that span the range over which the polarity is expected to flip, including the locations we tested here and those used by Hernandez et al. (2007). The results could help to pinpoint the location of auditory cortices, or at least the orientation of a prospective AEP dipole projecting from the cortical surface to the skin surface (described as “tilted to the rostroventral-dorsocaudal direction” by Supin et al. 2001).

The latency of a potential originating from a cortical source to anywhere on the skin surface is very similar, given conductance times for electrical signals. However, the way in which potentials from different generators in the cortex sum at the skin surface can alter peak values as well as peak latencies in the summed electrical activity; moreover, these effects differ, depending on electrode location (Luck 2014). In the present data, the temporal window that Hernandez et al. (2007) used to quantify P50 and N75 magnitudes resulted in a lower mean magnitude response at the 20 cm non-inverting electrode site than a temporal window of equal length that was shifted earlier in time. Electrode location is likely an important factor in explaining this difference; however, latency differences at the same electrode site can also occur due to idiosyncratic differences between dolphins (such as differences in brain geometry, or attentiveness). Determining the optimal temporal window over which to extract component peaks requires additional research using a greater number of non-inverting electrode locations and testing additional dolphins.

We observed that a 50-Hz low-pass filter yielded a nominal increase in SNRL in our measures ($^\sim 1$ dB). While often useful as a pre-processing stage for ICA, low-pass filtering may not be appropriate for all ACR studies, as AEPs were detectable even without filtering. If filtering is not used and SNRLs are smaller, it is likely to be more important to summarize AEP magnitudes by calculating the mean over a window centered around the expected peak time, rather than a local max or minima; specifically, noise at frequencies higher than the dominant frequencies of an ERP component can have a greater impact on peak values than on means (Luck 2014). This further underscores the need to better understand latency response variability for components such as the N1 and P2, and how this may vary across individual dolphins.

We observed a stimulus offset response, approximately 25 ms after the end of the stimulus, that follows the N1–P2 peak structure described for human offset AEPs (Hillyard and Picton 1978). The offset response was present in the sensor-space recordings from the meatus reference, as well as in the third ICA component from the dorsal and meatus references. This response was previously observed in dolphins with skull surface electrodes in response to a 500-ms duration pure tone and FM sweep (Ridgway 1980). The offset response was not reported by Hernandez et al. (2007), which may reflect differences in how strong the offset response is for different electrode montages. It may also be explained by differences in stimulus duration, as Hernandez et al. (2007) used 100-ms duration stimuli; in humans, the offset response strength increases as stimulus duration increases (Hillyard and Picton 1978) . The ACR offset response in humans is a form of an acoustic change complex (ACC), or response to a change in ongoing sound, such as a transition from a tone to white noise (Martin and Boothroyd 1999). This kind of ACC could be useful in probing how/where specific sound sequences or transitions are processed by the dolphin cortex.

### Conclusions

By comparing responses measured in different reference electrode montages, we find that a meatus electrode served as the best reference for sensor-space AEP measures, whereas a dorsal electrode served as the best reference for component-space measures, in terms of largest SNRL and peak-to-peak measures. Given that the dorsal reference montage introduced significant heartbeat contamination, complicating monitoring of the recording progress in sensor space, the utility of a dorsal reference might be limited in practice. Both sensor- and component-space AEP measures and SNRL estimates nominally benefited from a 50-Hz low-pass filter in the pre-processing stage. The use of a meatus reference is consistent with many previous ABR recordings, and may provide advantages in terms of building on prior literature to examine the time course of an AEP from brainstem to cortical potentials. These are important considerations, as the choice of the reference electrode has not previously been discussed thoroughly for dolphin AEPs. The selection is important to avoid or mitigate artifacts from cardiac or other myogenic sources. It should also be comparable to previously published literature (for further discussion, see Luck 2014). To effectively build upon this and prior work, further experiments should be conducted with the same electrode montages. Future multi-electrode studies should include a non-inverting sensor placed just posterior to the blowhole, as reported for mid- and long-latency AEPs (Hernandez et al. 2007) and short-latency brainstem responses (Finneran et al. 2019), in addition to one placed 20–30 cm posterior to the blowhole, as recommended from the current results, and supported by previous findings (Supin et al. 2001; Popov and Supin 1986).

All measures reported here were from a dolphin in-air and lying on a foam mat. AEPs collected in dolphins submerged in seawater cannot be unequivocally compared to the current results. Underwater recording of ACRs presents unique challenges, including increased muscle artifacts from movement of the dolphin to stabilize itself against ocean waves, and maintenance of a consistent distance and orientation between its head and a sound projector. The saltwater also acts as an electrical volume conductor that shunts current away from the dolphin’s head (Supin et al. 2001). Furthermore, ambient noise while dolphins are underwater might confound the interpretation of ACRs, at least in testing environments like San Diego Bay. All of these challenges will need to be addressed to move toward the study of dolphin auditory cognition in a more naturalistic situation.

## Supplementary Information

Below is the link to the electronic supplementary material.

Supplementary material 1 (pdf 2858 KB)

## Abbreviations

ABR: Auditory brainstem response
ACC: Acoustic change complex
ACR: Auditory cortical response
AEP: Auditory-evoked potential
ECG: Electrocardiogram
EEG: Electroencephalography
ERP: Event-related potential
IC: Independent component
ICA: Independent component analysis
SNR: Signal-to-noise ratio
SNRL: Signal-to-noise ratio level
SPL: Sound pressure level

## Footnotes

1 For an example of heartbeat contamination in a stacked time series, see supplementary Fig. 1a). Note also the relative similarities between the dorsal referenced first IC (Fig. 4a) and the high-amplitude fluctuations in the dorsal-referenced sensor-space counterpart in Fig. 2.
