# Supplementary material for: Measuring auditory cortical responses in Tursiops truncatus
Source: J Comp Physiol A Neuroethol Sens Neural Behav Physiol. 2021 Jul 30;207(5):629–40. doi: 10.1007/s00359-021-01502-5 (PMC8408064; doi:10.1007/s00359-021-01502-5)
Supplement: Supplementary file 1 — Supplementary material 1 (pdf 2858 KB) [file 359_2021_1502_MOESM1_ESM.pdf]

Supplementary Results

Table 1 Post-hoc sensor-space ERP N1-P2 component magnitude and peak-to-peak amplitudes using shifted temporal windows, 33-53 ms for N1 and 58-78 ms for P2 (µV).

	**Filtered**					**Unfiltered**				
reference	*N1 peak*	*N1 mean*	*P2 peak*	*P2 mean*	*Peak−Peak*	*N1 peak*	*N1 mean*	*P2 peak*	*P2 mean*	*Peak−Peak*
melon 20 *cm*	3.50	2.45	8.60	5.44	12.11	4.48	2.68	7.69	5.39	12.17
meatus 20 *cm*	2.35	1.64	5.36	3.40	7.70	2.90	2.00	4.92	3.17	7.81
meatus 30 *cm*	1.16	0.13	8.48	6.25	9.63	2.14	1.38	7.24	5.33	9.38

Table 2 Pairwise component metrics for activation residual validity test. Smaller values indicate greater similarity between two given components. Comparisons were not performed on different components from the same data set and are denoted by '-'

		**Comp 1**			**Comp 2**			**Comp 3**		
		Full	*Odd*	*Even*	*Full*	*Odd*	*Even*	*Full*	*Odd*	*Even*
	Full	0.00	0.01	0.01	-	4.68	4.54	-	61.52	77.77
**Comp 1**	*Odd*	0.01	0.00	0.01	7.07	-	4.82	76.80	-	76.15
	Even	0.01	0.01	0.00	7.20	5.06	-	77.22	60.61	-
	Full	-	7.07	7.20	0.00	0.05	0.07	-	11.19	12.93
**Comp 2**	*Odd*	4.68	-	5.06	0.05	0.00	0.01	20.14	-	19.21
	Even	4.54	4.82	-	0.07	0.01	0.00	20.52	16.48	-
	Full	-	76.80	77.22	-	20.14	20.52	0.00	0.02	0.01
**Comp 3**	*Odd*	61.52	-	60.61	11.19	-	16.48	0.02	0.00	0.02
	Even	77.77	76.15	-	12.93	19.21	-	0.01	0.02	0.00

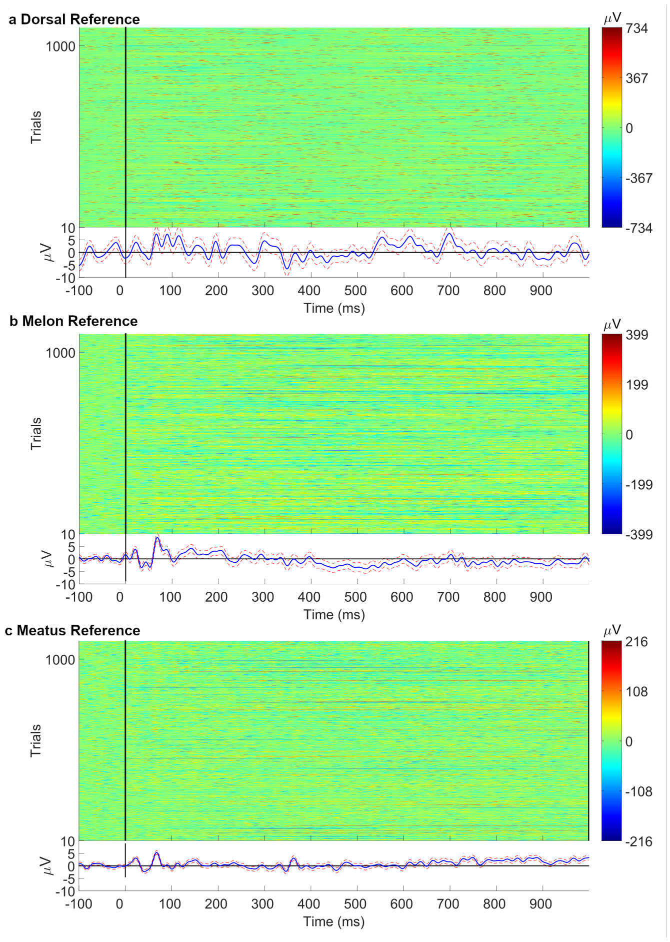

Fig. 1 Low-pass filtered ERP images from the non-inverting electrode 20 cm posterior of the blowhole and referenced to three locations: a. Dorsal b. Melon c. Meatus. Each ERP image contains a stacked series of epochs, from 1 to 1100. Voltage is represented by color, with scale on the right in microvolts (note different Y-scales for each reference recording average). Below the stacked series is the mean waveform with dash representing the standard error of the mean.

Table 3 Pairwise component metrics for cosine distance validity test. Smaller values indicate great similarity vis a vis a smaller cosine distance between two given components. Comparisons were not performed on different components from the same data set and are denoted by '-'

		Comp 1			Comp 2			Comp 3		
		Full	*Odd*	*Even*	*Full*	*Odd*	*Even*	*Full*	*Odd*	*Even*
**Comp 1**	*Full*	0.0	0.00	0.00	-	0.05	0.05	-	0.46	0.81
	Odd	0.00	0.0	0.00	0.08	-	0.05	0.67	-	0.81
	Even	0.00	0.00	0.0	0.08	0.05	-	0.66	0.46	-
**Comp 2**	*Full*	-	0.08	0.08	0.00	0.01	0.01	-	0.69	0.94
	Odd	0.05	-	0.05	0.01	0.00	0.01	0.84	-	0.99
	Even	0.05	0.05	-	0.01	0.01	0.00	0.83	0.63	-
**Comp 3**	*Full*	-	0.67	0.66	-	0.84	0.83	0.00	0.03	0.01
	Odd	0.46	-	0.46	0.69	-	0.63	0.03	0.00	0.07
	Even	0.81	0.81	-	0.94	0.99	-	0.01	0.07	0.00

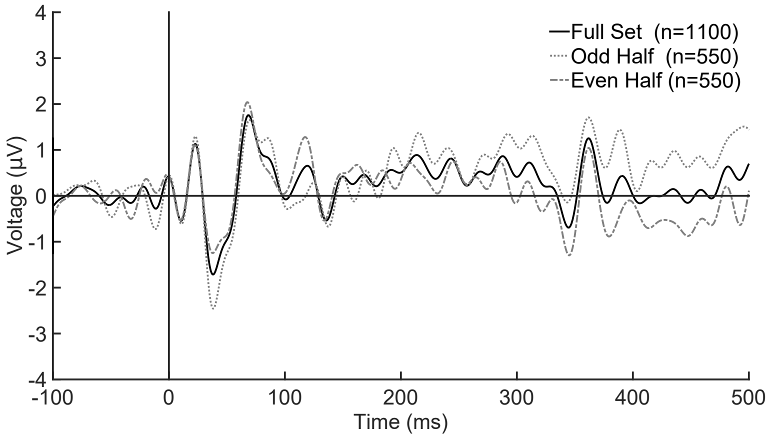

Fig. 2 Split half comparison of the dorsal montage third IC. The relative similarity between full and data set halves implies reliability of the ICA decomposition. The number of epochs in each data set is expressed by 'n' in the legend.
